# The Lithium-Ion Battery Recycling Trilemma: Bridging the Gap Between Material Science, Economic Reality, and Regulatory Policy

**DOI:** 10.3390/ma19061235

**Published:** 2026-03-20

**Authors:** Qi Zhang

**Affiliations:** 1 BCMaterials, Basque Center for Materials, Applications and Nanostructures, UPV/EHU Science Park, 48940 Leioa, Spain; qi.zhang@bcmaterials.net; 2 IKERBASQUE, Basque Foundation for Science, Plaza Euskadi, 5, 48009 Bilbao, Spain

**Keywords:** lithium-ion battery recycling, circular economy, critical materials, policy, supply chain resilience

## Abstract

The electric vehicle revolution has created an urgent need for lithium-ion battery (LIB) recycling, with projections exceeding 11 million tons of end-of-life batteries annually by 2030. However, progress toward a circular economy remains fragmented. This perspective article introduces the concept of a ‘Recycling Trilemma,’ arguing that technological advancements in material separation are systematically undermined by economic volatility and regulatory fragmentation. While current literature focuses on isolated domains—chemistry, business models, or policy—this work provides a systems-level synthesis. By analyzing the friction points between material science (e.g., binder removal, impurity sensitivity), economic realities (e.g., logistics costs, LFP profitability), and regulatory frameworks (e.g., EU vs. US divergence), we propose that true circularity requires synchronized design-for-recycling, market stabilization mechanisms, and harmonized digital product passports. The paper concludes that overcoming the trilemma demands a shift from isolated fixes to integrated, cross-sectoral coordination.

## 1. Introduction

It is increasingly apparent that the electric vehicle revolution is about to encounter significant systemic friction. We are staring down a tsunami of spent batteries, with projections suggesting over 11 million tons will need managing every year by 2030 [1,2,3,4,5,6]. The scale of the challenge is well-documented [7,8], with projections of an annual end-of-life stream exceeding two million metric tons of battery packs by 2030, creating an urgent economic and environmental imperative for scalable recycling solutions. This presents a critical paradox [9]. Lithium-ion batteries (LIBs) are crucial for cleaning up our air, but their “make-use-discard” lifecycle threatens to swap exhaust pollution for a significant environmental and resource security risk of resource depletion and toxic waste. The environmental implications of battery production and disposal [10] encompass significant carbon footprints from raw material extraction and risks of soil and water contamination from improper end-of-life handling.

If you follow the academic literature, you would think we are close to a solution. The focus has been laser-like on process chemistry, tweaking leaching formulas or regenerating cathode materials. This work is important, but it misses the bigger picture. This narrow focus is symptomatic of a broader systemic fragmentation, where technological research, economic modeling, and circular business development proceed in isolation [9], missing the interconnected reality. There is a vast and frustrating gap between a 99% pure recovery in a controlled lab and a profitable, functioning recycling plant. That beautiful chemistry often falls apart when faced with the messy reality of thousands of different battery types, wildly swinging metal prices, and a patchwork of conflicting regulations. Recent reviews [11] highlight the gap between lab-scale success and the industrial-scale implementation of recycling technologies, where factors like feedstock variability, logistical costs, and economic viability present formidable barriers.

**Figure 1:**
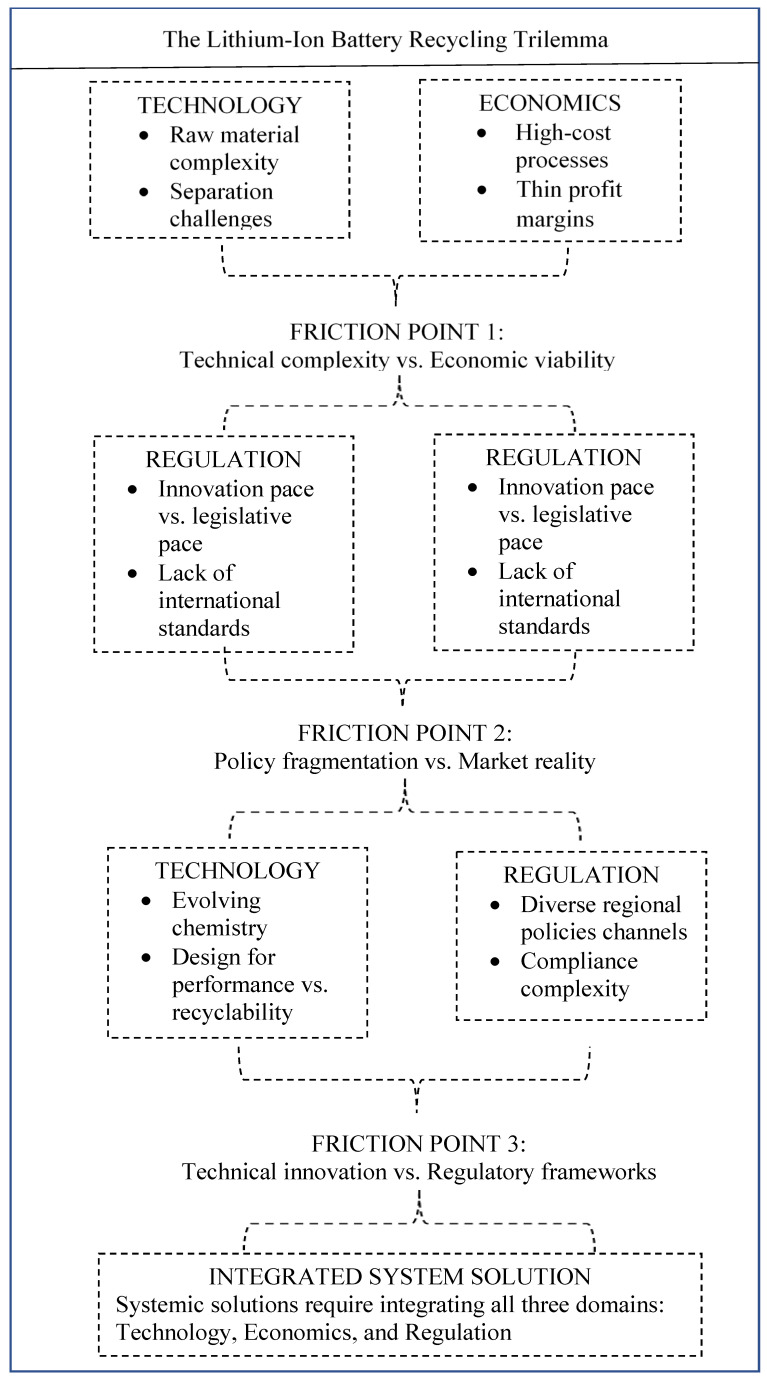
The lithium-ion battery recycling trilemma.

This article proceeds from a simple, if uncomfortable, premise: the industry is trapped in a recycling trilemma. Progress in one area—technology, economics, or regulation—is too often undone by constraints in another. For example, a brilliant new cell design that boosts energy density (a tech win) might be impossible to recycle economically (an economic loss). Similarly, well-intentioned, strict transport rules (a policy win) can make the most efficient mega-recycling plant too expensive to supply. The following analysis unpacks these tensions and proposes a pathway to transcend them, moving from the fragmented state of the trilemma (Figure 1) toward an integrated, circular system. The aim is not merely to catalog problems, but to sketch a framework for a genuinely systemic solution.

To contextualize the novelty of this framework, it is instructive to compare it against the existing literature that has shaped the field. While previous works have provided invaluable insights into specific domains, namely, the technical intricacies of recycling processes or the strategic dimensions of circular business models, they have largely treated these domains in isolation. A synthesis that explicitly maps the interactions and frictions between material science, economic viability, and regulatory policy remains absent. Table 1 positions the proposed ‘Trilemma’ framework against two of the most influential recent reviews, highlighting the distinct systems-level perspective this work aims to contribute.

This comparison underscores that while technical progress is essential, it is insufficient without a synchronized evolution of economic structures and regulatory mandates—a central thesis this perspective article will explore in the following sections.

This perspective article proceeds by dissecting each vertex of the trilemma, technology, economics, and regulation, in turn. Through this analysis, we will argue that no single domain can advance in isolation; rather, the path forward requires a coordinated realignment. Specifically, we will demonstrate that design-for-recycling innovations (Section 2) must be paired with decentralized logistics models (Section 3) and enabled by digital infrastructure and harmonized policy (Section 4). The synthesis presented in Section 5 outlines how these elements can converge to transform the current linear ‘black box’ into a truly circular system.

## 2. The Technological Dimension: The Material Science of Separation

Here is the inconvenient truth: the hardest part of recycling is not extracting metals, it is separating the battery pack in the first place. Today’s battery packs are engineering marvels, optimized for safety, performance, and longevity. Unfortunately, that optimization often makes them a recycler’s significant processing challenge.

### 2.1. The Binder Problem and Design for Disassembly

Take the humble binder. At the electrode level, the industry standard is Polyvinylidene fluoride (PVDF). It is fantastic at its job, chemically inert and gluing the active material firmly to the foil current collector. For recyclers, however, PVDF is a significant impediment. Getting the valuable material off the foil requires brute-force methods: intense heat (which wastes energy and creates emissions) or nasty, toxic solvents like NMP.

There is hope in water-soluble or alternative binders [12], such as carboxymethyl cellulose or polyacrylic acid derivatives, which have shown promise in lab-scale recycling studies [13,14,15]. Recent advances in water-soluble binders, such as those reviewed by Trivedi et al. [13], offer a pathway to avoid PVDF entirely, though challenges remain regarding long-term electrochemical stability in high-voltage cathodes [16]. Alternative polymer composites offer pathways to more recyclable electrode designs [11,16]. The paradigm shift toward recyclable design, including “debondable-on-demand” concepts, is now recognized as a critical research frontier [4]. However, industry adoption is slow, held back by concerns over long-term stability in the cell. What we really need is a paradigm shift toward “debondable-on-demand” adhesives. Imagine a glue that holds fast for a decade but lets go on command at the end of the battery’s life when triggered by a specific signal. That is the kind of clever design we should be chasing.

### 2.2. The Limits of Direct Recycling

Direct recycling is the holy grail—the idea of refurbishing the cathode material without dissolving it. Direct recycling methods have shown promise in regenerating cathode materials [17]. In theory, it is the most energy-efficient path. In practice, it hits a brick wall called heterogeneity. Cathode chemistry is a moving target, evolving from nickel-manganese-cobalt (NMC) 111 to 622, 811, and beyond. Toss all these different versions into one recycling stream, and you get a useless, middling-grade material. This requires a quantum leap in automated sorting and characterization—an area where advances in AI and robotics are emerging but need significant development to handle real-world heterogeneity [7,18,19,20].

### 2.3. Impurity Profiles in Hydrometallurgy

Wet chemistry (hydrometallurgy) is the industry workhorse, great for recovery rates but notoriously finicky about impurities. Copper and aluminum from shredded packs dissolve right alongside the prized nickel and cobalt, creating a purification headache that drives up cost. Comprehensive reviews of hydrometallurgical challenges [10,21,22,23] confirm its acute sensitivity to feedstock impurities, making the purification of valuable metals a major cost and complexity driver in recycling operations. For example, Davis and Demopoulos [24] recently demonstrated that even trace amounts of copper (>0.1%) in recycled NMC811 can trigger voltage fade and impedance growth, effectively downgrading the material’s value below virgin-grade specifications. This sensitivity intensifies as cathodes move toward higher nickel content [25].

## 3. The Economic Dimension: Logistics and Valuation Models

### 3.1. The Logistics Tax and Class 9 Hazards

Spent lithium-ion batteries are classified as Class 9 hazardous materials due to their inherent electrochemical instability and potential for thermal runaway. This material hazard—arising from residual charge and reactive electrolytes—dictates stringent handling and transport protocols. Consequently, moving a spent battery is not simply a logistics problem; it is a safety-critical operation requiring specialized certified packaging, trained drivers, and approved routes.

Much of the materials science literature glosses over a critical factor: the “logistics tax.” A spent lithium-ion battery is not just scrap, it is a Class 9 hazardous good, a potential inherently hazardous energy storage device requiring specialized handling. Transporting it requires special certified packaging, trained drivers, and approved routes. This safety imperative is not optional, and comprehensive logistics analyses confirm it can devour 40–50% of total recycling costs [2,7,26], a critical factor frequently omitted or underestimated in economic models [26]. This single fact undermines the entire concept of a centralized “mega-refinery.” A more realistic model is decentralized: local “spoke” facilities shred batteries into stable, less hazardous “black mass,” which is then shipped to centralized “hub” refineries. The economics demand this structural shift. While a full techno-economic assessment of this decentralized architecture is beyond the scope of this Perspective, the logic is grounded in established cost drivers. As modeled by Schlott et al. [26], the fixed costs of compliance (specialized containers, insurance) are scale-independent, making long-distance transport of full cells prohibitive. Conversely, the chemical refining process (hub) benefits from economies of scale. Shredding at the spoke transforms the material state, reducing its hazard classification and thereby decoupling logistics costs from refining efficiency.

### 3.2. The LFP Conundrum

The industry’s growing love affair with Lithium Iron Phosphate (LFP) chemistry is a ticking time bomb for recyclers. LFP batteries are great for cost and safety, but they contain no high-value cobalt or nickel. Often, the raw material value inside a used LFP cell is less than the cost of recycling it. This flips the script entirely. This economic inversion transforms recycling from ‘urban mining’ to ‘waste management,’ creating a fundamental challenge for circular business models [5,27]. Recycling Nickel-Manganese-Cobalt (NMC) batteries is “urban mining.” Recycling LFP batteries is “waste management”, a cost center, not a revenue source. Without policy tools like Extended Producer Responsibility (EPR) that mandate and fund their recycling, mountains of Lithium Iron Phosphate (LFP) batteries will likely be stockpiled or, worse, landfilled.

### 3.3. Commodity Volatility

Building a multi-million-dollar recycling plant on the shifting sands of lithium and cobalt prices is economically unsustainable, as the 2023–24 price correction demonstrated. Lithium carbonate prices, for instance, fell by over 80% from their peak in late 2022, dropping from approximately $83,000/ton to below $15,000/ton by early 2024, while cobalt prices similarly contracted by over 25% amid persistent oversupply [28]. This volatility undermines investment, as detailed in several techno-economic analyses of recycling facilities [10,29]. To attract serious capital, this industry needs stability: long-term offtake agreements and price floors that de-risk the massive upfront investment. We cannot build a critical circular infrastructure on commodity speculation. This inherent financial instability prevents the long-term capital investment required to build the recycling infrastructure that the energy transition demands.

## 4. The Regulatory and Geopolitical Dimension

Policy sets the rules of the game, but right now, the rulebook is fragmented and full of contradictions.

### 4.1. The “Black Mass” Geopolitical Trap

There is a glaring strategic inconsistency in Western critical mineral strategy. We are pouring billions into building domestic battery “Gigafactories” while outsourcing the dirty work of recycling. Lacking domestic refining capacity, we ship our shredded battery waste—”black mass”—to Asia. It is a strategic counterproductive outcome: we keep the waste but export the mineral value and the associated jobs. True sovereignty requires policies that support the entire value chain, including the complex chemical refining most Western nations currently avoid. Strategic analyses highlight this vulnerability in Western critical mineral supply chains [8].

### 4.2. Divergent Strategies: EU vs. US

The transatlantic divide is instructive. The EU is taking a hardline “compliance push” with its new battery regulation, mandating recycled content levels. This creates an artificial, policy-driven market. The US, via the Inflation Reduction Act, prefers an “incentive pull,” using tax credits to boost domestic supply. Both have merit, but for global companies, this divergence is a compliance headache that stifles efficiency. This regulatory divergence creates compliance complexity that may hinder the development of efficient global recycling ecosystems [6,9].

### 4.3. The Digital Imperative

Recycling in the dark does not work. Today, a recycler receives a pallet of black boxes and they have no efficient way to know what is inside. This forces them to use inefficient, one-size-fits-all processes. The Battery Digital Product Passport (DPP) is not just a nice idea, it is a foundational necessity. Such digital infrastructure is increasingly recognized as foundational for circular systems [3]. A DPP that details chemistry, construction, and health history would enable precise sorting and optimal processing, slashing costs and boosting output quality overnight.

## 5. Pathways Forward: Integrating the System

**Figure 2:**
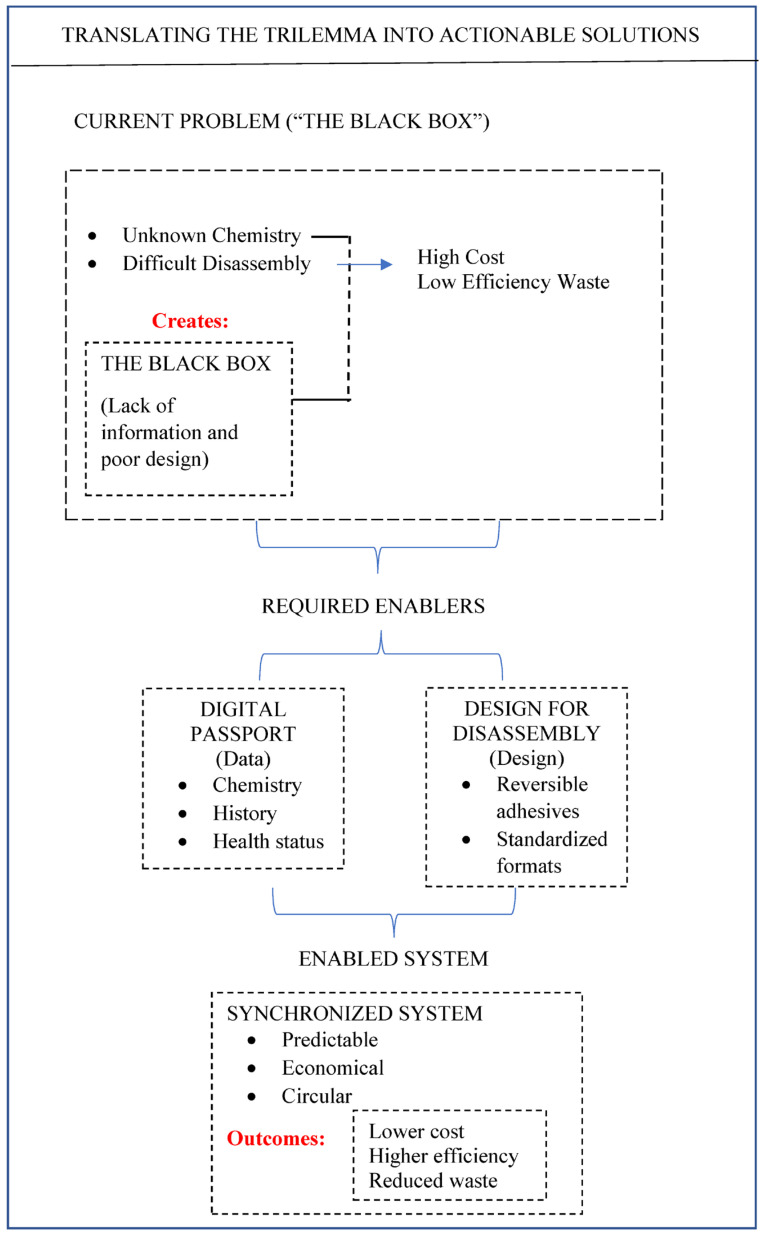
Translating the trilemma into actionable solutions. The pathway from the current problematic “black box” to an enabled, synchronized system is driven by two key enablers: Digital Product Passport (data) and Design for Disassembly (design).

To escape the trilemma, we must connect the dots and enable the paradigm shift outlined in Figure 2: from a problematic ‘black box’ to a predictable, circular system.

This transition is predicated on two concurrent enablers: data transparency and physical accessibility. With these in place, three integrated pathways become viable. Overcoming the trilemma requires policy that acts not just on one vertex, but on the interactions between them. For instance, subsidies for domestic refining (addressing economics) must be paired with design standards (addressing technology) that ensure batteries are actually recyclable. Furthermore, the implementation of Extended Producer Responsibility (EPR) schemes can internalize the logistics costs that currently make LFP recycling unprofitable, transforming a ‘waste management’ cost into a funded circular obligation.

- Co-Design for Circularity: Battery engineers and recyclers need integrated collaboration from day one. Studies show design choices lock in ~80% of environmental impact [4,9,30,31], making standardized formats and reversible adhesives more critical than marginal energy density gains.
- Regionalized Closed Loops and Second-Life Pathways: Co-locating recycling hubs with gigafactories reduces transportation emissions and enhances supply chain security [7,8,32]. This geographical synergy is also crucial for enabling the reuse of retired EV batteries in local energy storage systems, a strategy shown by life-cycle assessment to significantly improve environmental outcomes before final recycling [32]. Integrated system analyses therefore support such regionalized models [7,32] by demonstrating that co-locating pre-processing with refining hubs minimizes the high costs and risks of transporting hazardous battery waste over long distances. Policy must streamline permitting for domestic refining capacity to enable these loops.
- Holistic Value Recognition: The business case must account for externalities and systemic benefits. Life-cycle assessments quantify the carbon reduction and environmental savings of recycling over primary mining [33,34], while economic models highlight the supply chain security value [9,33,35].

## 6. Conclusions

The lithium-ion battery stands as a monument to materials science ingenuity, yet its afterlife exposes the fragility of linear industrial models. By framing the recycling challenge as a ‘trilemma’—where technology, economics, and regulation exert conflicting pressures—this perspective article has sought to demonstrate that no single breakthrough will suffice. The binder problem (Tech) cannot be solved without addressing the cost of separation (Econ). The LFP profitability gap (Econ) cannot be closed without mandates (Reg) that value waste reduction over raw material arbitrage. The strategic inconsistency of exporting black mass (Reg) undermines the very supply chain resilience we seek to build.

The path forward lies in synchronization. Designers must adopt ‘debondable-on-demand’ principles, enabling low-cost separation. Regulators must harmonize digital product passports to provide the data transparency recyclers desperately need. Economists and investors must look beyond spot prices to recognize the strategic value of domestic critical mineral security. As outlined in Figure 2, the transition from a problematic ‘black box’ to a predictable circular system is achievable, but only if we treat the trilemma not as three separate problems, but as one interconnected system requiring an integrated response.

## Figures and Tables

**Table 1:** Comparison of the scope and novelty of the present work with key prior reviews in the field.

Reference	Primary Focus	Identified Gap/Novelty Contribution
Harper et al. (2019) [7]	Comprehensive technical review of recycling processes, including pyrometallurgy, hydrometallurgy, and direct recycling.	Treats economics and policy as contextual background factors rather than as interconnected constraints that actively shape technological outcomes.
Wesslkämper and von Delft (2024) [9]	Circular business models and corporate strategy for electric vehicle battery recycling.	Focuses on firm-level value capture and strategic innovation, but does not address the systemic tension between material science limitations and regulatory frameworks.
This Work	The “Trilemma” Framework: Demonstrates how progress in one domain (Technology, Economics, or Regulation) is actively undermined by constraints in the other two.	Provides a systems-level perspective bridging the three silos; emphasizes the need for synchronized design-for-recycling, market stabilization mechanisms, and harmonized policy feedback loops.

## Data Availability

No new data were created or analyzed in the study. Data sharing is not applicable to this article.
